# Curculigoside Protects against Titanium Particle-Induced Osteolysis through the Enhancement of Osteoblast Differentiation and Reduction of Osteoclast Formation

**DOI:** 10.1155/2021/5707242

**Published:** 2021-07-04

**Authors:** Fangbing Zhu, Jianyue Wang, Yueming Ni, Wei Yin, Qiao Hou, Yingliang Zhang, Shigui Yan, Renfu Quan

**Affiliations:** ^1^Department of Orthopaedic Surgery, Second Affiliated Hospital, School of Medicine, Zhejiang University, Hangzhou, 310009 Zhejiang Province, China; ^2^Department of Orthopaedics, Xiaoshan Traditional Chinese Medical Hospital, Hangzhou, 311201 Zhejiang Province, China; ^3^Department of Orthopedics, Affiliated Jiangnan Hospital of Zhejiang Chinese Medical University, Hangzhou, 311201 Zhejiang Province, China; ^4^School of Medicine, Zhejiang University, Hangzhou, 310058 Zhejiang Province, China

## Abstract

Wear particle-induced periprosthetic osteolysis is mainly responsible for joint replacement failure and revision surgery. Curculigoside is reported to have bone-protective potential, but whether curculigoside attenuates wear particle-induced osteolysis remains unclear. In this study, titanium particles (Ti) were used to stimulate osteoblastic MC3T3-E1 cells in the presence or absence of curculigoside, to determine their effect on osteoblast differentiation. Rat osteoclastic bone marrow stromal cells (BMSCs) were cocultured with Ti in the presence or absence of curculigoside, to evaluate its effect on osteoclast formation *in vitro*. Ti was also used to stimulate mouse calvaria to induce an osteolysis model, and curculigoside was administrated to evaluate its effect in the osteolysis model by micro-CT imaging and histopathological analyses. As the results indicated, in MC3T3-E1 cells, curculigoside treatment attenuated the Ti-induced inhibition on cell differentiation and apoptosis, increased alkaline phosphatase activity (ALP) and cell mineralization, and inhibited TNF-*α*, IL-1*β*, and IL-6 production and ROS generation. In BMSCs, curculigoside treatment suppressed the Ti-induced cell formation and suppressed the TNF-*α*, IL-1*β*, and IL-6 production and F-actin ring formation. *In vivo*, curculigoside attenuated Ti-induced bone loss and histological damage in murine calvaria. Curculigoside treatment also reversed the RANK/RANKL/OPG and NF-*κ*B signaling pathways, by suppressing the RANKL and NF-*κ*B expression, while activating the OPG expression. Our study demonstrated that curculigoside treatment was able to attenuate wear particle-induced periprosthetic osteolysis in *in vivo* and *in vitro* experiments, promoted osteoblastic MC3T3-E1 cell differentiation, and inhibited osteoclast BMSC formation. It suggests that curculigoside may be a potential pharmaceutical agent for wear particle-stimulated osteolysis therapy.

## 1. Introduction

It is no doubt that total joint arthroplasty (TJA) is a landmark of orthopedic surgery in the 20th century, with the notable advantages of joint pain relief and joint function restoration in patients with bone and articular maladies [1]. A follow-up study of more than 15 years reported that approximately 82% of total knee replacements (TKRs) last 25 years and 70% of unicondylar knee replacements (UKRs) last 25 years; the 25-year pooled survival rate of hip replacements and joint replacement was 77.6% and 57.9%, respectively, indicating generally excellent outcomes of TJA [2, 3]. However, all TJA will eventually fail because of the gradually emerged issues, such as infection, fracture, or loosening and wear. And these factors are the most prevalent reasons for TJA revision. Among them, the ratio of aseptic loosening (38%) is higher than the sum of deep infection and periprosthetic fractures, and most of the aseptic loosening is caused by particle wear and thereby induced osteolysis [4, 5]. Thus, osteolysis has become a key challenge restricting the life of artificial joint and is also the main cause of TJA renovation.

In an artificial joint, wear particles accumulate around the prosthesis over the years, aggravate local friction between the bone and implant, and further increase wear particle accumulation [6]. On the other hand, the accumulated wear particles could stimulate the secretion of inflammatory mediators, like NF-*κ*B, IL-1*β*, TNF-*α*, and IL-6, to induce chronic inflammation and periprosthetic osteolysis. During this process, RANKL/RANK/OPG and NF-*κ*B signaling pathways play an important role. NF-*κ*B could be activated by macrophages due to the phagocytosis of wear-produced particles, which then regulates the expression of other mediators, such as TNF-*α*, IL-1*β*, and IL-6 [7]. These mediators are particularly involved in the activation and differentiation of osteoclasts, to mediate osteoclastic bone resorption and lead to osteolysis [6, 8]. RANKL (receptor activator of nuclear factor *κ*B ligand), a critical osteoclast differentiation factor, is massively secreted by periprosthetic fibroblasts, osteoblasts, and stromal cells. It combines with RANK and cooperates with NF-*κ*B, to promote osteoclast differentiation and survival, as well as osteoclastic bone resorption [9]. However, OPG, an osteoclast differentiation inhibitor, usually competes with RANK to integrate RANKL and blocks the promotion effect of RANKL/RANK on osteoclast and osteoclastic bone resorption, to inhibit osteolysis. As a result, the imbalance between osteoblastic bone formation and osteoclastic bone resorption ultimately induces osteolysis of the periprosthesis. Hence, a therapeutic agent focusing on promoting osteogenesis and preventing osteolysis could be a promising strategy for osteolysis of the periprosthesis.

*Curculigo orchioides* Gaertn. is a medicinal herb used for the treatment of lumbar and knee joint arthritis for a long history in traditional Chinese medicine [10, 11]. As a major compound of *Curculigo orchioides*, curculigoside also proves profound bone-protective activity. *In vivo* and *in vitro* studies reported that curculigoside exhibits antiarthritic effects in a rat model of arthritis and rheumatoid arthritis-derived MH7A cells [12]. Ding et al. reported that curculigoside significantly relieved the hind paw swelling and arthritis index to protect against adjuvant arthritis in model rats [13]. Curculigoside was also found to alleviate excess iron overload-induced bone loss in Zhang et al.'s study [14]. These findings indicated that curculigoside is beneficial for osteogenesis and plays a protective role in bone and articular maladies. Therefore, we hypothesized the inhibition potential of curculigoside on osteolysis induced by wear particles in TJA. In this study, titanium particles (Ti) were chosen to mimic periprosthetic osteolysis in *in vivo* and *in vitro* studies; then, the effect of curculigoside on osteolysis was investigated. Taken together, our results hope to provide curculigoside as a potential therapeutic agent for the prevention and treatment of osteolysis induced by wear particles.

## 2. Materials and Methods

### 2.1. Titanium Particle Preparation

In this study, osteolysis was stimulated using titanium particles (Ti), which were purchased from ALFA. Over 95% of the Ti diameters were ≤4 *μ*m. Then, titanium particles underwent the following procedures to remove endotoxin. Ti were calcined at 180°C for 6 h and further soaked in concentrated hydrochloric acid for 5 h. After centrifugation to remove concentrated hydrochloric acid, Ti were rinsed in 75% ethanol for 48 h. The concentration of endotoxins was detected using a chromogenic end-point TAL on a diazo coupling detection kit, and endotoxin concentration < 0.1 EU/ml was qualified. Then, DMEM was added to obtain Ti suspension at a concentration of 0.1 mg/ml for subsequent experiments [15].

### 2.2. Osteoblast Culture and Differentiation Induction

The mouse osteoblast cell line MC3T3-E1 was purchased from iCell Bioscience Inc. (Shanghai, China) and maintained in Alpha-Minimum Essential Medium (*α*-MEM) containing 10% FBS in a humidified atmosphere of 5% CO_2_ at 37°C. The cell differentiation was induced by using cultured DMEM supplemented with 10% FBS, 1% penicillin/streptomycin, 100 nM dexamethasone, 10 mM *β*-glycerophosphate, and 50 mM vitamin C [16]. And Ti (0.1 mg/ml) were added into the cells to stimulate periprosthetic osteolysis and inhibit osteoblast differentiation in MC3T3-E1 cells. Then, MC3T3-E1 cells were treated with curculigoside (HPLC purity ≥ 98%, EY-B0626, Shanghai Yiyan Biotechnology Co. Ltd., China) at 25, 50, and 100 *μ*g/ml in the presence of Ti.

### 2.3. Bone Marrow Stromal Cell (BMSC) Isolation and Treatment

Healthy C57/BL6 mice aged 4-6 weeks were obtained from Shanghai Slack Animal Co., Ltd. (Shanghai, China) and housed in a temperature of 22~26°C, humidity of 70%, and 12 h light/dark cycle, with free access to water and feeding. All animal procedures were approved by the Animal Research Committee of Zhejiang Traditional Chinese Medical University (Hangzhou, China). The BMSCs were isolated from the tibias and femurs of mice and incubated with *α*-MEM containing 30 ng/ml M-CSF, 10% FBS, and 1% penicillin/streptomycin to induce mature osteoclast. The medium was changed every three days. Then, 5 × 10^5^ BMSCs were seeded in 12-well plates containing *α*-MEM supplemented with 0.1 mg/mL Ti. And curculigoside at increasing concentrations (25, 50, and 100 *μ*g/ml) was added into the cells with the presence of Ti.

### 2.4. Cell Viability Assay

MC3T3-E1 cells or BMSCs in the logarithmic phase were seeded in 96-well plates and coincubated with curculigoside for 24 h, 48 h, or 72 h. After that, the CCK-8 reagent (10 *μ*l, MCE) was added into each well for incubation for another 2 h. Then, the optical density was measured using a microplate reader (CMaxPlus, MD) at a wavelength of 450 nm to evaluate cell viability.

### 2.5. Cell Apoptosis Assay

Cell apoptosis in each group was assessed using flow cytometry (FCM). Briefly, logarithmic phase MC3T3-E1 cells were seeded in 6-well plates, and binding buffer was added into the plates and centrifuged to remove the supernatants. After being remixed with 100 *μ*l binding buffer, cells were maintained with annexin V-FITC (5 *μ*l, CoWin Biosciences, China) and PI (10 *μ*l, CoWin Biosciences, China) for reaction in the dark room for 15 min. Afterwards, binding buffer (400 *μ*l) was added into the plates, and cell apoptosis was assessed by FCM (C6, BD Biosciences, USA).

### 2.6. Alkaline Phosphatase (ALP) Assay

The ALP assay was performed to evaluate the osteogenic function of MC3T3-E1 cells. After cells were treated with curculigoside (25, 50, and 100 *μ*g/ml) in the presence of Ti, cells were further maintained in osteogenic induction medium for 7 days. Then, an ALP kit (Beyotime, China) was used for ALP staining according to the manufacturer's protocol, and p-nitrophenyl phosphate (pNPP) (Sigma, USA) served as a substrate. Afterwards, cells were observed under light microscopy (AE2000, Motic, China) at 405 nm.

### 2.7. Alizarin Red Staining

Cell mineralization was evaluated using Alizarin Red S staining. After being stimulated with the indicated agents (curculigoside, Ti), MC3T3-E1 cells were fixed in 4% paraformaldehyde for 15 min. Next, cells were stained with 500 *μ*l/well Alizarin Red solution (Solarbio, China) for 20 min at room temperature. Finally, cells were ringed 3 times with ddH2O and imaged under an inverted fluorescence microscope (Ts2-FC, Nikon, Japan).

### 2.8. Mitochondrial Membrane Potential Assay

The mitochondrial membrane potential was analyzed using a 5,5,6,6-tetrachloro-1,1,3,3-tetraethyl-imidacarbocyanine iodide (JC-1) probe. Briefly, MC3T3-E1 cells were seeded in 6-well plates with 3 × 10^5^ cells/well and intervened with curculigoside and Ti. Then, cells were added with 1 ml of JC-1 staining solution (Beyotime, China) per well for incubation for 20 min at 37°C under dark. At the end of incubation, the fluorescence density of the stained cells was monitored under FCM (C6, BD Biosciences, USA).

### 2.9. Intracellular ROS Measurement

After MC3T3-E1 cells were intervened with curculigoside and Ti, cell culture medium was replaced with a ROS indicator, 2′,7′-dichlorodihydrofluoresceindiacetate (DCF-DA), for 30 min at 37°C. After incubation, the fluorescence was detected by FCM (C6, BD Biosciences, USA).

### 2.10. Enzyme-Linked Immunosorbent Assay (ELISA)

After MC3T3-E1 cells or BMSCs were intervened with curculigoside and Ti, cells were added into 96-well plates and maintained at 37°C for 48 h. After that, cells were centrifuged to obtain the supernatants. Then, the cell supernatants were filtered through a 0.22 *μ*m filter and detected with the corresponding ELISA kits including TNF-*α*, IL-1*β*, IL-6, RANKL, and OPG (MEIMIAN, China) according to the manufacturer's protocol. Finally, the optical density was detected by using a microplate reader (CMaxPlus, Molecular Devices, USA) at 450 nm.

### 2.11. Immunofluorescence Assay of F-actin Ring Formation

BMSCs were grown on cover glasses and treated with curculigoside and Ti. After that, 1 ml of 0.5% Triton X-100 was added into each well for cell permeabilization. And 3% BSA was used to block the reaction; then, BMSCs were incubated with the primary antibody against F-actin (Abcam, USA) overnight at 4°C and further incubated with the secondary antibody (Abcam, USA). After incubation and washing with PBS, the cell nuclei were stained with DAPI. Cells were observed under an inverted fluorescence microscope (Ts2-FC, Nikon, Japan).

### 2.12. RT-qPCR

The total RNA of MC3T3-E1 cells or BMSCs was extracted using a TRIzol reagent (Sangon Biotech, China) and reverse transcribed into cDNA with the cDNA Synthesis kit (CW Bioscience, China) following the manufacturer's protocol. RT-qPCR analysis was performed using a Roche LightCycler® 96 real-time PCR system. The following conditions were used: 95°C, 10 min; 95°C, 15 s; 60°C, 60s; and 40 cycles. The primers used are listed in Table 1, and GAPDH was used as an internal control. The gene expression was analyzed using the comparative 2^−ΔΔCq^ method.

### 2.13. Western Blot Assay

The total proteins of MC3T3-E1 cells or BMSCs were extracted and lysed with RIPA buffer (Beyotime, China), and protein concentration was detected using a BCA protein assay kit (Solarbio, China). Then, a 20 *μ*g total protein sample was loaded onto 10% sodium dodecyl sulfate polyacrylamide gel electrophoresis (SDS-PAGE) for separation and transferred to a PVDF membrane. After being blocked with 5% skimmed milk, the membrane was incubated at 37°C with the primary antibodies against caspase-3 (ab13847, Abcam), caspase-9 (ab32539, Abcam), SIRT1 (ab110304, Abcam), BMP-2 (ab214821, Abcam), RANKL (ab45039, Abcam), OPG (ab73400, Abcam), I*κ*B*α* (ab32518, Abcam), p-I*κ*B*α* (AF2002, Affinity), p65 (ab207297, Abcam), p-p65 (AF2006, Affinity), IKK*α* (ab32041, Abcam), p-IKK*α* (AF3013, Affinity), NFATc1 (ab2796, Abcam), and cathepsin K (ab19027, Abcam) at 4°C overnight. After that, the membrane was further incubated with the secondary antibody at room temperature for 1 h. Then, the enhanced chemiluminescence (ECL) reagent (Solarbio, China) was used to visualize the protein bands, and the relative band density was semiquantified with the ImageJ software.

### 2.14. Animal Experiments and Drug Administration

Male C57/BL6 mice were randomly divided into four groups with 8 rats per group: sham group, Ti group, curculigoside 20 mg/kg group, and curculigoside 40 mg/kg group. A Ti-induced osteolysis model in mouse calvaria was prepared as previously described [14, 16]. After the mice were anesthetized and fixed on a sterile operating table, the skin of the head was disinfected with skin disinfectant; then, in the middle of the calvaria, an incision was made. And 20 mg Ti containing PBS was gently injected to the anterior fontanelle of the skull [16]. The sham group was injected with the same volume of PBS without Ti. After the incision was stitched up with 4-0 surgical sutures, mice were turned back into the cages with conventional feeding. On 2 days, mice in the curculigoside group were injected (i.p.) with curculigoside at a dosage of 20 mg/kg/d and 40 mg/kg/d, respectively. Sham and Ti groups were injected with saline instead. After 14-day administration, mice in each group were sacrificed, and the calvaria tissues were collected for further experiments.

### 2.15. Micro-CT Analysis

A micro-CT (MCT-III, ZKKS) was applied to detect the surface erosion in the calvaria of each group of mice before sacrifice. The scanning parameters were set as follows: voltage 45 keV, current 555 *μ*A, power 25 W, exposed time 48 ms, resolution 35 *μ*m, scanning angle 180°, and scanning thickness 9 *μ*m with continuous scanning. Volume of interest (VOI) was determined in the center of each calvaria. The CTvox software was used for 3D reconstruction and data processing. Bone mineral density (BMD), bone volume (BV), ratio of bone volume to tissue volume (BV/TV), and trabecular thickness (Tb.Th) were determined.

### 2.16. HE Staining and TRAP Staining

The obtained calvaria tissues were fixed and embedded in molten paraffin. Then, tissues were sectioned into 4 *μ*m pieces and stained with hematoxylin and eosin (H&E) and tartrate-resistant acid phosphatase (TRAP) according to the manufacturer's protocols. Images were obtained with a light microscope (DM3000, Leica, Germany).

### 2.17. Immunohistochemical (IHC) Analysis

The calvaria tissue sections were dewaxed with xylene and subjected to gradient hydration and antigen retrieval with citric acid solution. Sections were added with corresponding primary antibodies against RANKL, OPG, TNF-*α*, IL-1*β*, and IL-6 and incubated overnight at 4°C. Then, IHC-positive stained cells were stained using a DAB Horseradish Peroxidase Color Development Kit and photographed using a light microscope (DM3000, Leica, Germany).

### 2.18. Statistical Analysis

Data were expressed as means ± standard deviation (SD). Statistical significance among multiple comparisons was analyzed by one-way ANOVA with the SNK test. A value of *P* < 0.05 was considered statistically significant.

## 3. Results

### 3.1. Curculigoside Attenuated the Ti-Induced Inhibition of Osteoblastic Differentiation in MC3T3-E1 Cells

**Figure 1:**
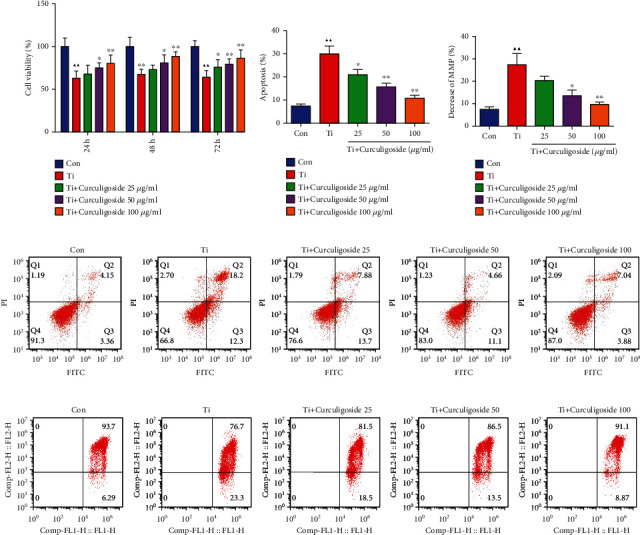
Curculigoside treatment attenuated Ti-induced inhibition of osteoblastic differentiation in MC3T3-E1 cells. (a) After being intervened with Ti and treated with curculigoside at different concentrations (25, 50, and 100 *μ*g/ml) for 24 h, 48 h, and 72 h, MC3T3-E1 cell viability was detected by the CCK-8 assay (*n* = 6). (b, d) After being intervened with Ti and treated with curculigoside, cell apoptosis was detected by flow cytometry (*n* = 3). (c, e) Mitochondrial membrane potential of MC3T3-E1 cells cultured under Ti and different concentrations of curculigoside was assayed by flow cytometry using JC-1 assay kits, and the decrease in the MMP index in each group cell was quantified (*n* = 3). Results are represented as the mean ± SD. ^▲^*P* < 0.05, compared to the Con group; ^▲▲^*P* < 0.01, compared to the Con group; ^∗^*P* < 0.05, compared to the Ti group; ^∗∗^*P* < 0.01, compared to the Ti group. Ti: titanium particle; Con: control; MMP: mitochondrial membrane potential.

After osteoblast MC3T3-E1 cells were intervened with Ti and treated with curculigoside at different concentrations (25, 50, and 100 *μ*g/ml) for 24 h, 48 h, and 72 h, cell viability was detected by the CCK-8 assay. As displayed in Figure 1(a), compared to the Con group, the cell viability of Ti group cells was significantly inhibited in 24 h, 48 h, and 72 h. While being compared to the Ti group, the cell viability in curculigoside-treated groups was increased, with significant differences in curculigoside 50 and 100 *μ*g/ml groups in 24 h, 48 h, and 72 h treatment and curculigoside 25 *μ*g/ml groups in 48 h and 72 h treatment. FCM results in Figures 1(b) and 1(d) showed that after Ti intervention, the MC3T3-E1 cell apoptosis rate was significantly increased compared to the Con group, while in curculigoside-treated groups at different concentrations, cell apoptosis was decreased.

### 3.2. Curculigoside Treatment Alleviated Ti-Induced Mitochondrial Damage in MC3T3-E1 Cells

As shown in Figures 1(c) and 1(e), the FCM results showed that after being intervened with Ti, the mitochondrial membrane potential was reduced in MC3T3-E1 cells. However, curculigoside treatment offset the decrease in the mitochondrial membrane potential, especially in curculigoside 50 and 100 *μ*g/ml groups.

### 3.3. Curculigoside Treatment Alleviated Ti-Induced Osteogenic Reduction and Osteoblastic Mineralization Inhibition in MC3T3-E1 Cells

**Figure 2:**
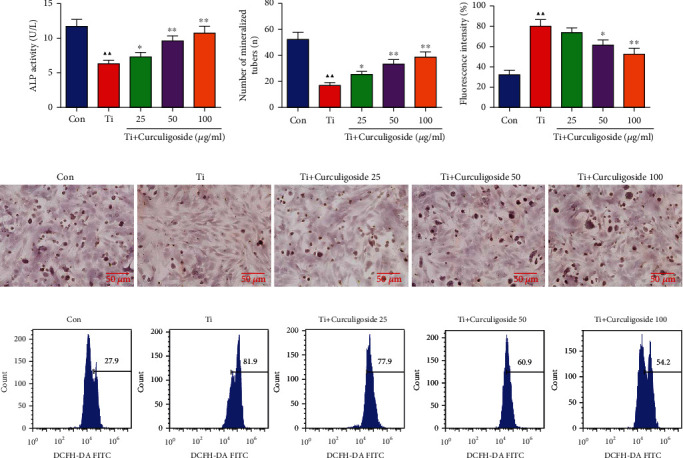
Curculigoside treatment alleviated Ti-induced inhibition on ALP activity, mineralization, and ROS generation in MC3T3-E1 cells. (a) MC3T3-E1 cells were detected with ALP staining, and ALP activity was quantified. (b, d) MC3T3-E1 cells were assayed by Alizarin Red staining, and the mineralization in each group cell was quantified. (c, e) Intracellular ROS level of MC3T3-E1 cells after Ti intervention and curculigoside treatment was assayed by flow cytometry, and ROS production in each group cell was quantified (*n* = 3); results are represented as the mean ± SD. ^▲^*P* < 0.05, compared to the Con group; ^▲▲^*P* < 0.01, compared to the Con group; ^∗^*P* < 0.05, compared to the Ti group; ^∗∗^*P* < 0.01, compared to the Ti group. Ti: titanium particle; Con: control; ALP: alkaline phosphatase.

ALP results demonstrated that Ti intervention significantly inhibited ALP activity compared to the Con group, which indicated an inhibitory effect of Ti on osteoblastic differentiation (Figure 2(a)). However, this osteogenic inhibition effect was reversed by curculigoside treatment; it recovered the ALP activity in MC3T3-E1 cells. Consistent with this, Alizarin Red staining showed that curculigoside treatment effectively stimulated cell mineralization (Figures 2(b) and 2(d)). And the number of mineralized tubes was also significantly increased in curculigoside-treated cells.

### 3.4. Curculigoside Treatment Inhibited the ROS Generation in Ti-Intervened MC3T3-E1 Cells

Intracellular ROS generation results in Figures 2(c) and 2(e) showed that compared with the Con group, the ROS generation was significantly increased after Ti intervention. However, in curculigoside-treated cells, the increased ROS generation induced by Ti was decreased, which indicated an oxidative stress inhibition effect of curculigoside in MC3T3-E1 cells.

### 3.5. Osteoblast-Associated Factors and Proinflammatory Cytokine Levels in MC3T3-E1 Cells

**Figure 3:**
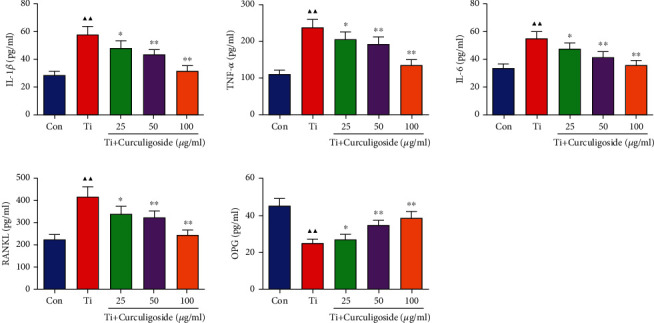
The levels of TNF-*α*, IL-1*β*, IL-6, RANKL, and OPG in MC3T3-E1 cells were detected using ELISA kits (*n* = 3); results are represented as the mean ± SD. ^▲^*P* < 0.05, compared to the Con group; ^▲▲^*P* < 0.01, compared to the Con group; ^∗^*P* < 0.05, compared to the Ti group; ^∗∗^*P* < 0.01, compared to the Ti group. Ti: titanium particle; Con: control.

The levels of TNF-*α*, IL-1*β*, IL-6, RANKL, and OPG in MC3T3-E1 cells were detected (Figure 3). It could be observed that compared to the Con group, the levels of proinflammatory cytokines in MC3T3-E1 cells, including TNF-*α*, IL-1*β*, and IL-6, were significantly increased with Ti intervention. However, in curculigoside-treated groups, these increased levels were restored and decreased significantly compared to the Ti group. As a differentiation factor of osteoclasts, the increased level of RANKL in Ti-intervened cells was significantly decreased with curculigoside treatment. Corresponding to this, the decreased level of OPG, an osteoclast differentiation suppressor, in Ti-intervened cells was significantly increased with curculigoside treatment.

### 3.6. Curculigoside Treatment Promoted the Osteoblast- and Apoptosis-Associated Gene Expression in Ti-Induced MC3T3-E1 Cells

**Figure 4:**
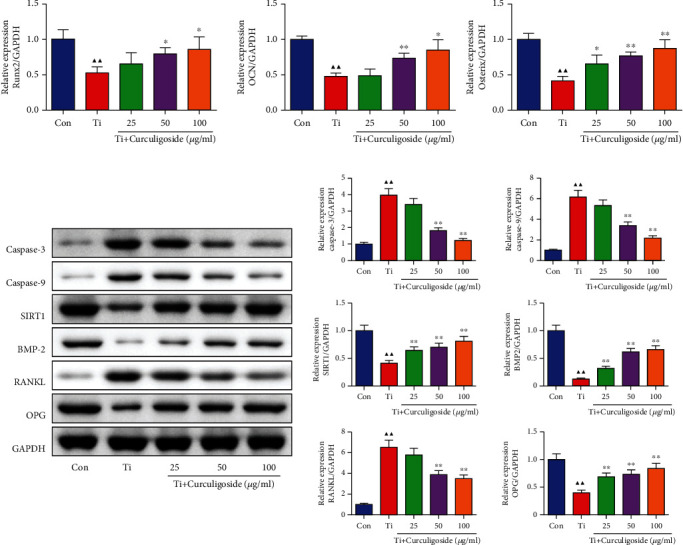
Curculigoside treatment promoted the osteogenic-associated gene expression in Ti-induced in MC3T3-E1 cells. (a) qRT-PCR analysis was performed to detect the relative mRNA expression of Runx2, osterix, and OCN in MC3T3-E1 cells. (b) Western blot analysis was performed to detect the relative protein expression of casepase-3, casepase-9, SIRT1, BMP-2, RANKL, and OPG in MC3T3-E1 cells (*n* = 3); results are represented as the mean ± SD. ^▲^*P* < 0.05, compared to the Con group; ^▲▲^*P* < 0.01, compared to the Con group; ^∗^*P* < 0.05, compared to the Ti group; ^∗∗^*P* < 0.01, compared to the Ti group. Ti: titanium particle; Con: control.

qRT-PCR results demonstrated that Ti intervention in MC3T3-E1 cells induced a significant inhibition on the expression levels of osteoblastic differentiation-related genes, including Runx2, OCN, and osterix (Figure 4(a)). Curculigoside treatment resulted in a significant increase in the expression levels of Runx2, OCN, and osterix. As shown in Figure 4(b), the relative protein levels of caspase-3, caspase-9, and RANKL were significantly increased in Ti group cells compared with the untreated Con group; SIRT 1, BMP-2, and OPG were significantly decreased in Ti group cells, while curculigoside treatment offset the protein expression of these genes.

### 3.7. Curculigoside Treatment Inhibited the Ti-Induced Activation of Osteoclast Activity in BMSCs

**Figure 5:**
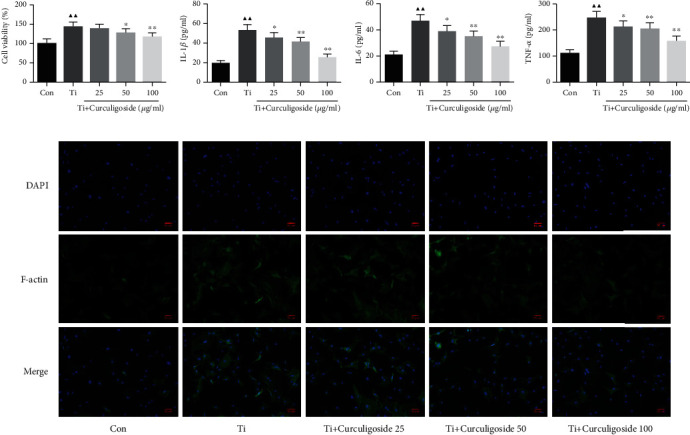
Curculigoside treatment inhibited Ti-induced activation of osteoclast activity in BMSCs. (a) After being intervened with Ti and treated with curculigoside at different concentrations (25, 50, and 100 *μ*g/ml) for 48 h, BMSC viability was detected by the CCK-8 assay (*n* = 6). (b) The levels of TNF-*α*, IL-1*β*, and IL-6 in BMSCs were detected using ELISA kits. (c) Cellular immunofluorescence of F-actin in BMSCs (*n* = 3); results are represented as the mean ± SD. ^▲^*P* < 0.05, compared to the Con group; ^▲▲^*P* < 0.01, compared to the Con group; ^∗^*P* < 0.05, compared to the Ti group; ^∗∗^*P* < 0.01, compared to the Ti group. BMSCs: bone marrow stromal cells; Ti: titanium particles; Con: control.

BMSCs were also intervened with Ti to induce osteoclast activity, and CCK-8 results in Figure 5(a) showed that cell viability in Ti group BMSCs was significantly increased compared to that in the Con group. However, in curculigoside 50 and 100 *μ*g/ml group BMSCs, the cell viability was decreased significantly compared to the Ti group. Ti treatment also significantly increased the levels of TNF-*α*, IL-1*β*, and IL-6 in BMSCs, and curculigoside treatment restored the elevated levels of these procytokines (Figure 5(b)). And for bone resorption-related prerequisite, F-actin, its expression intensity enhanced by Ti was diminished by curculigoside administration (Figure 5(c)).

### 3.8. Curculigoside Treatment Alleviated Osteoclast-Associated Gene Expression in Ti-Induced BMSCs

**Figure 6:**
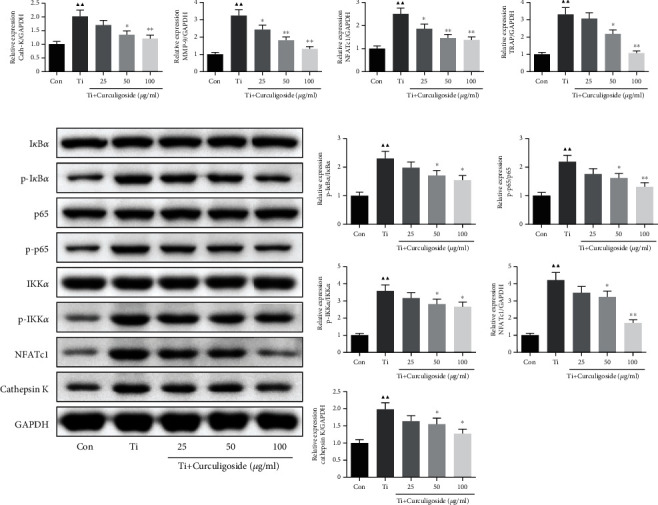
Curculigoside treatment alleviated osteoclast-associated gene expression in Ti-induced BMSCs. (a) qRT-PCR analysis was performed to detect the relative mRNA expression of cathepsin K, TRAP, NFATc1, and MMP-9 in BMSCs. (b) Western blot analysis was performed to detect the relative protein expression of I*κ*B*α*, p-I*κ*B*α*, p65, p-p65, IKK*α*, p-IKK*α*, NFATc1, and cathepsin K in BMSCs (*n* = 3); results are represented as the mean ± SD. ^▲^*P* < 0.05, compared to the Con group; ^▲▲^*P* < 0.01, compared to the Con group; ^∗^*P* < 0.05, compared to the Ti group; ^∗∗^*P* < 0.01, compared to the Ti group. BMSCs: bone marrow stromal cells; Ti: titanium particles; Con: control.

qRT-PCR results in Figure 6(a) showed that the mRNA expressions of cath-K, TRAP, NFATc1, and MMP-9 were significantly increased in BMSCs with Ti intervention. And in Ti-intervened BMSCs incubated with curculigoside, the expressions of these genes were restrained. Ti also induced the increased protein expressions of p-I*κ*B*α*, p-p65, p-IKK*α*, NFATc1, and cathepsin K in BMSCs (Figure 6(b)). And in curculigoside-treated groups, the protein expressions of p-I*κ*B*α*/I*κ*B*α*, p-p65/p65, p-IKK*α*/IKK*α*, NFATc1, and cathepsin K were all decreased.

### 3.9. Curculigoside Treatment Attenuated Ti-Induced Osteolysis and Enhanced Bone Formation in Model Mice

**Figure 7:**
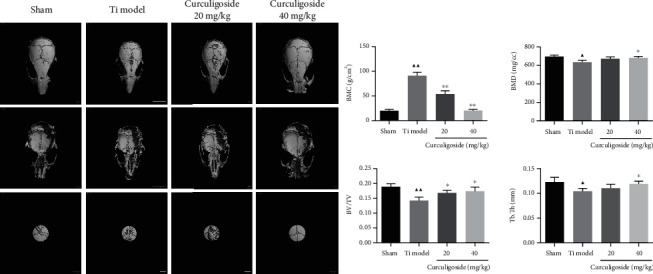
Curculigoside treatment attenuated Ti-induced osteolysis in a mouse calvaria model. (a) Micro-CT reconstruction images of the calvaria in each group of mice. (b) BMC, BMD, BV/TV, and Tb.Th were determined (*n* = 3); results are represented as the mean ± SD. ^▲^*P* < 0.05, compared to the sham group; ^▲▲^*P* < 0.01, compared to the sham group; ^∗^*P* < 0.05, compared to the Ti group; ^∗∗^*P* < 0.01, compared to the Ti group. Ti: titanium particle; BMC: bone mineral content; BMD: bone mineral density; BV/TV: bone volume to tissue volume; Tb.Th: trabecular thickness.

In *in vivo* experiments of the Ti-induced osteolysis mouse model, micro-CT results (Figure 7) illustrated that Ti injection induced osteolysis and bone loss in mouse calvaria. The osteolysis-related parameters, including BMC, BMD, BV/TV, and Tb.Th, in the Ti group were also significantly changed compared to those in the sham group. While curculigoside treatment attenuated the morphological alteration and osteolysis of calvaria in Ti model mice, the relative parameters were also attenuated.

### 3.10. Curculigoside Treatment Restored Histological Damage of the Calvaria

**Figure 8:**
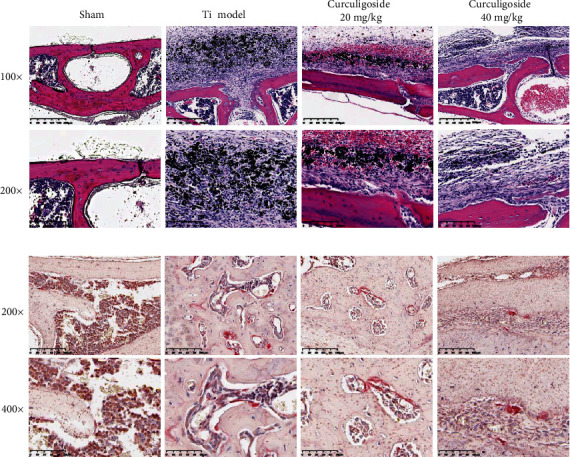
Curculigoside treatment attenuated Ti-induced osteolysis in histological analysis of calvaria sections: (a) HE staining; (b) TRAP staining. Ti: titanium particle.

HE and TRAP staining was performed to analyze the histological changes of the calvaria in Ti model mice upon curculigoside treatment. HE staining results (Figure 8(a)) showed that the calvaria in Ti group mice showed osteolysis characteristics, with eroded bone surface observed. TRAP staining (Figure 8(b)) also showed that the TRAP-positive cells were increased in Ti model group mouse calvaria. In curculigoside treatment groups, the degree of Ti-induced osteolysis and TRAP-positive cells were attenuated.

### 3.11. Curculigoside Treatment Attenuated Ti-Induced Osteolysis by Enhancing Osteogenesis

**Figure 9:**
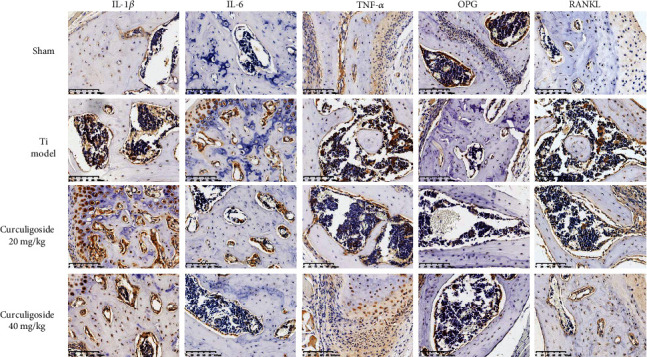
Immunohistochemical analysis for RANKL, OPG, TNF-*α*, IL-1*β*, and IL-6 in calvaria sections of each group of mice. Ti: titanium particle. Magnification, ×200.

The IHC results in Figure 9 demonstrated that Ti injection increased the expression of IL-1*β*, IL-6, RANKL, and TNF-*α* and inhibited OPG expression in the calvaria of mice, to increase the proinflammatory cytokine production and the RANKL/OPG ratio. While after curculigoside treatment, the expressions of IL-1*β*, IL-6, RANKL, and TNF-*α* were inhibited, OPG was increased, thus restoring the RANKL/OPG ratio and inhibiting inflammation.

## 4. Discussion

Joint prosthesis longevity and function are largely dependent on the degree of interface wear between the bone and implant, as well as the formation pace of wear particles. Against this, many strategies, including new types of prosthesis, wear-resistant materials, surgical methods, fixation techniques, and rehabilitation management improvements, have been developed and applied in TJA [17–21]. These strategies greatly avoid joint prosthesis loosening and wear, but wear still cannot be solved completely. Wear particles came to be an unavoidable problem that leads to periprosthetic osteolysis and TJA failure. As mentioned before, wear particles could induce macrophages and osteoclasts to secrete proinflammatory and osteoclastogenic cytokines and break the balance between the bone formation by osteoblasts and the bones resorption by osteoclasts, thus resulting in periprosthetic osteolysis.

Osteoblastic bone formation and osteoclastic bone resorption are critically responsible for the maintenance of bone homeostasis [22]. Osteoblast serves as a functional cell for bone formation and participates in the synthesis, secretion, and mineralization of the bone matrix. Thus, the proliferation and differentiation of osteoblasts directly affect osteogenesis, and abnormalities of these physiologies could lead to bone diseases, including osteoporosis, osteoarthritis, and primary bone tumors [23, 24]. Increasing evidences show that wear particles significantly affect osteoblastic physiologies and suppress bone formation, to induce osteolysis. Particles inhibit the osteoblast's proliferation, and inhibiting calcium deposition and ALP activity suppressed the osteoblast's ability to produce a mineralized bone matrix [25]. In addition, the particles are capable of stimulating osteoblasts to secrete proinflammatory cytokines. A study about Ti-stressed murine MC3T3-E1 cells reported that Ti reduced osteoblast differentiation by inhibiting ALP activity, matrix mineralization, and osteogenesis-related gene expression, as well as increasing the RANKL/OPG ratio in cells [26]. In this study, our results also indicated an inhibition role of Ti in osteoblast differentiation and osteogenesis. Results showed that Ti intervention significantly decreased MC3T3-E1 cell viability and promoted the production of TNF-*α*, IL-1*β*, and IL-6, cell apoptosis, and apoptotic gene expression, including caspase-3 and caspase-9. More importantly, ALP staining showed that the activity of ALP, a marker of osteoblast differentiation, was inhibited; Alizarin Red staining showed that cell mineralization was inhibited with Ti treatment. Osteogenic-associated gene expression was also changed in Ti-intervened MC3T3-E1 cells, including RANKL, OPG, Runx2, osterix, and OCN.

Osteoclast is a significant functional cell for bone resorption and also has a critical role in wear particle-induced periprosthetic osteolysis. *In vivo*, these wear particles have the ability to activate the proinflammatory cells, gather at the periprosthetic area, and activate osteoclasts for bone resorption; *in vitro*, these particles also activate osteoclast formation and differentiation, to stimulate bone resorption [27–29]. In this study, we isolated BMSCs from rats to investigate the effect of Ti on osteoclasts. Corresponding with published studies, our results showed that Ti stimulated osteoclast viability and the production of TNF-*α*, IL-1*β*, and IL-6 and increased F-actin ring formation, a cytoskeleton structure of osteoclasts. In addition, NF-*κ*B and related NFATc1 pathway are also involved in osteoclast formation effect of Ti, and the expression of NF-*κ*B p65, I*κ*B*α*, cathepsin K, TRAP, NFATc1, and MMP-9 was changed in Ti-intervened BMSCs.

In this study, *in vitro* experiments showed that curculigoside reversed the Ti-induced inhibition of osteoblast-associated bone formation in MC3T3-E1 cells and activation of osteoclast-associated bone resorption in BMSCs, to inhibit osteolysis. In MC3T3-E1 cells, curculigoside treatment reversed the Ti-induced inhibition of cell differentiation, increased ALP activity and cell mineralization, and inhibited apoptosis, inflammation, and ROS generation. In BMSCs, curculigoside treatment suppressed the Ti-induced cell activity and suppressed the inflammatory cytokines and F-actin ring formation. The expressions of osteoblast- and osteoclast-associated genes were also restored by curculigoside. The osteogenic induction effect of curculigoside was also found in normal BMSCs, represented by increased ALP activity, cell mineralization, and expression of osteogenic genes (ALP, Col I, OCN, OPG, and Runx2) [30]. And curculigoside's major metabolite in rat plasma, M2, was also found to exhibit antiosteoporotic activity in osteoblastic MC3T3-E1 cells [14]. In Ti-induced osteolysis in mouse calvaria, relative results also showed that curculigoside treatment was able to attenuate bone loss, suggesting its potential for the treatment of wear particle-induced periprosthetic osteolysis. In Wang et al.'s study [31], they also demonstrated that curculigoside was able to alleviate bone loss in mice and MC3T3-E1 cells, the biochemical parameters related to bone metabolism and the expression of Runx2 and OCN were improved by curculigoside, and the bone-mineralized nodule formation was also increased *in vitro*, which were consistent with our findings. In arthritic rats, a study also proved that curculigoside treatment decreases serum levels of TNF-*α*, IL-1*β*, IL-6, IL-10, IL-12, and IL-17A and regulated the expression of NF-*κ*B p65 and I*κ*B, to exhibit significant antiarthritic effects [11]. Therefore, combined with these studies, we suggested the inhibition potential of curculigoside on osteolysis induced by wear particles.

Furthermore, we explored the potential molecular mechanism of curculigoside's positive impact on osteogenesis. To our knowledge, many cytokines, hormones, genes, and signaling pathways are involved in bone remodeling [21]. The RANK/RANKL/OPG signal is critically correlated with periprosthetic osteolysis [32]. OPG, an osteoclastogenesis inhibitory factor, can bind to RANKL to block RANKL/RANK, and this further affects the main signaling pathways of osteoclast differentiation and activation. RANK/RANKL/OPG signaling also regulates osteoclast formation, activation, and survival via modulating different signaling pathways, including I*κ*K*α*/NF-*κ*B and calcineurin/NFATc1 [33, 34]. Researches focus on these signals also providing clues for developing therapeutic approaches for bone diseases, including osteolysis [35–37]. As mentioned earlier, previous studies also demonstrated the regulation potential of curculigoside on RANK/RANKL/OPG and NF-*κ*B signaling pathways [11, 13, 14]. And in this study, the results showed that curculigoside reversed the RANK/RANKL/OPG and NF-*κ*B signaling pathway-associated gene expression in Ti-induced osteolysis *in vitro* and *in vivo*, by inhibiting RANKL, NF-*κ*B p-p65/p65, p-I*κ*B*α*/I*κ*B*α*, and p-IKK*α*/IKK*α* and increasing OPG.

## 5. Conclusion

In summary, based on *in vitro* and *in vivo* experiments, the present study demonstrated that curculigoside treatment was able to attenuate Ti-induced periprosthetic osteolysis, activate osteoblastic MC3T3-E1 cell differentiation, inhibit osteoclast BMSC formation, and enhance bone formation in the mouse model. And this protective effect was involved in the regulation of RANK/RANKL/OPG and NF-*κ*B signaling pathways. We propose that curculigoside may be a potential pharmaceutical agent used for the prevention and treatment of wear particle-stimulated osteolysis.

## Figures and Tables

**Table 1:** Primer sequences of the gene.

Gene	Forward primer	Reverse primer
Mouse Runx2	5′-TGGCCGGGAATGATGAGAAC-3′	5′-TGAAACTCTTGCCTCGTCCG-3′
Mouse osterix	5′-GTGGGAACAAGAGTGAGCTGG-3′	5′-CCATAGTGAGCTTCTTCCTGGGTA-3′
Mouse OCN	5′-CCTGAGTCTGACAAAGCCTTCA-3′	5′-AGATGCGTTTGTAGGCGGTC-3′
Mouse cath-K	5′-TTCCCGCAGTAATGACACCC-3′	5′-GGAACCACACTGACCCTGAT-3′
Mouse TRAP	5′-CTGCTGGTCATTCCTGTCGT-3′	5′-GCAGGGGGTAAGATCTCATT-3′
Mouse NFATc1	5′-TGGAGAAGGCTCCAGATGGC-3′	5′-CTGGTTGCGGAAAGGTGGTA-3′
Mouse MMP-9	5′-TTGAGTCCGGCAGACAATCC-3′	5′-ACTTCCAGTACCAACCGTCC-3′
Mouse GAPDH	5′-TGTCAAGCTCATTTCCTGGTATG-3′	5′-TTATGGGGGTCTGGGATGGA-3′

## Data Availability

All data generated or analyzed during this study are included in this article.
